# Ethyl 1-*O*-*tert*-butyl­dimethyl­silyl-2,3-*O*-isopropyl­idene-5-[(2′*S*)-tetra­hydro­pyran-2-yl­oxy]-d-*glycero*-α-d-*manno*-hepto­furonate

**DOI:** 10.1107/S1600536808021193

**Published:** 2008-07-12

**Authors:** Raquel G. Soengas, Laura Valencia, Juan C. Estévez, Ramón J. Estévez

**Affiliations:** a Departamento de Química Orgánica, Universidade de Santiago de Compostela, 15782 Santiago de Compostela, Spain; b Departamento de Química Inorgánica, Facultade de Química, Universidad de Vigo, 36310 Vigo, Pontevedra, Spain

## Abstract

The title compound {systematic name: (2*S*,3*R*)-ethyl 3-[(3a*S*,4*R*,6*S*,6a*S*)-6-*tert-*butyl­dimethyl­silyl­oxy-2,2-dimethyl­per­hydro­furo[3,4-*d*][1,3]dioxol-4-yl]-2-nitro-3-[(*S*)-tetra­hydro-2*H*-pyran-2-yl­oxy]propanoate}, C_23_H_41_NO_10_Si, is the product of the Henry reaction of 1-*O*-*tert*-butyl­dimethyl­silyl-2,3-*O*-isopropyl­idene-α-d-*lyxo*-penta­dialdo-1,4-furan­ose with ethyl nitro­acetate and the subsequent protection of its C-5 hydr­oxy group as tetra­hydro­pyranyl, in order to avoid the retro-Henry reaction. The tetra­hydro­pyranyl group adopts a chair conformation. The absolute configuration, assumed from the synthesis, was confirmed from the diffraction data.

## Related literature

For the preparation of the aldehyde precursor of the title compound, see: Brewster *et al.* (1987 ▶). For the Henry reaction, see: Soengas *et al.* (2003*a* ▶). For the protection as tetra­hydro­pyran, see: Soengas *et al.* (2003*b* ▶). For other related literature, see: Gruner *et al.* (2002 ▶); Lillelund *et al.* (2002 ▶); Ogawa *et al.* (2005 ▶); Chakraborty *et al.* (2002 ▶).
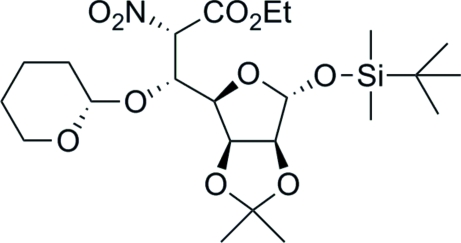


## Experimental

### 

#### Crystal data

C_23_H_41_NO_10_Si
*M* *_r_* = 519.66
Orthorhombic, 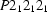

*a* = 15.593 (3) Å
*b* = 9.563 (4) Å
*c* = 19.690 (3) Å
*V* = 2935.9 (15) Å^3^
*Z* = 4
Cu *K*α radiation
μ = 1.13 mm^−1^
*T* = 293 (2) K
0.48 × 0.40 × 0.32 mm

#### Data collection

Enraf–Nonius TurboCAD-4 diffractometer
Absorption correction: ψ scan (North *et al.*, 1968 ▶) *T*_min_ = 0.614, *T*_max_ = 0.714
6473 measured reflections
5898 independent reflections
3434 reflections with *I* > 2σ(*I*)
*R*_int_ = 0.037
3 standard reflections every 167 reflections intensity decay: 4%

#### Refinement

*R*[*F* ^2^ > 2σ(*F* ^2^)] = 0.066
*wR*(*F* ^2^) = 0.264
*S* = 1.11
5898 reflections
325 parameters
H-atom parameters constrained
Δρ_max_ = 0.21 e Å^−3^
Δρ_min_ = −0.27 e Å^−3^
Absolute structure: Flack (1983 ▶), 2569 Friedel pairs
Flack parameter: 0.04 (8)

### 

Data collection: *CAD-4 EXPRESS* (Enraf–Nonius, 1994 ▶); cell refinement: *CAD-4 EXPRESS*; data reduction: *XCAD4* (Harms & Wocadlo, 1995 ▶); program(s) used to solve structure: *SIR97* (Altomare *et al.*, 1999 ▶); program(s) used to refine structure: *SHELXL97* (Sheldrick, 2008 ▶); molecular graphics: *ORTEP-3 for Windows* (Farrugia, 1997 ▶); software used to prepare material for publication: *WinGX* (Farrugia, 1999 ▶).

## Supplementary Material

Crystal structure: contains datablocks I, global. DOI: 10.1107/S1600536808021193/cf2203sup1.cif

Structure factors: contains datablocks I. DOI: 10.1107/S1600536808021193/cf2203Isup2.hkl

Additional supplementary materials:  crystallographic information; 3D view; checkCIF report

## Figures and Tables

**Fig. 1.**
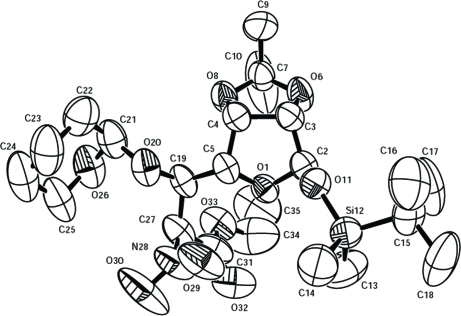
The molecular structure of (3), with atom labels and 50% probability displacement ellipsoids. H atoms have been omitted.

**Fig. 2.**
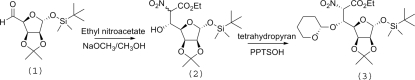
Chemical reaction scheme.

## supplementary crystallographic information

### Comment

Nitro-sugars are very important as precursors of a wide range of natural and synthetic products with relevant properties (Gruner *et al.*, 2002), as aminopoliols (Lillelund *et al.*., 2002; Ogawa *et al.*, 2005), polyhydroxylated amino acids (Chakraborty *et al.*., 2002), *etc*. The title nitro-sugar compound (3), C_23_H_41_NO_10_Si, is the product of the Henry reaction (Soengas *et al.*, 2003a) of 1-*O*-*tert*-butyldimethylsilyl-2,3-*O*-isopropylidene-a-*D*-*lyxo*-pentadialdo-1,4-furanose (1) (Brewster *et al.*, 1987) with ethyl nitroacetate to give ethyl 1-*O*-*tert*-butyldimethylsilyl-2,3-*O*-isopropylidene-D-*glycero*-a-D-*manno-*heptofuronate (2) and the subsequent protection of its C5 hydroxy group as tetrahydropyranyl (Soengas *et al.*, 2003b), in order to avoid the retro Henry reaction. The molecular structure is represented in Fig. 1. Bond lengths and angles are within the expected values. The tetrahydropyranyl group adopts a chair conformation. The absolute configuration was assumed from the synthesis and confirmed by the X-ray crystal structure determination.

### Experimental

Ethyl nitroacetate (0.26 ml, 2.36 mmol) and sodium methoxide (1.12 g, 2.28 mmol) were added to a solution of 1-*O*-*tert*-butyldimethylsilyl-2,3-*O*-isopropylidene-α-*D*-*lyxo*-pentadialdo-1,4-furanose (1) (0.62 g, 2.1 mmol) in dry methanol (6 ml), cooled at 273 K under argon. The reaction mixture was stirred at room temperature for 5 h and then neutralized with DOWEX 50W resin, filtered, evaporated *in vacuo*, and the resulting residue submitted to flash column chromatography (ethyl acetate/hexane 1:6) to give the epimeric mixture of ethyl 1-*O*-*tert*-butyldimethylsilyl-2,3-*O*-isopropylidene-D-*glycero*-a-D-*manno-*heptofuronate (2) (0.63 g, 70% yield) as an unstable gum.

Dry tetrahydropyran (0.39 ml, 4.32 mmol) and pyridinium *p*-toluenesulfonate (0.12 g, 0.43 mmol) were added to a solution of the above epimeric mixture (0.63 g, 1.45 mmol) in dry dichloromethane (12 ml). The solution was stirred at room temperature for 24 h, then diethyl ether (18 ml) was added and the mixture washed with brine (12 ml), dried with anhydrous sodium sulfate, filtered and evaporated *in vacuo* to give a residue that was purified by flash column chromatography (ethyl acetate/hexane 1:9) to give an oil; after crystallization from hexane this gave the title compound (3) (0.24 g, 32%) as a white crystalline solid. mp 369–370 K; [*a*]_D_^20^ +108.0° (*c*, 1 in CHCl_3_); IR (NaCl, cm^-1^) 1762 (C=O), 1569, 1373 (NO_2_); ^1^H NMR (300 MHz, CDCl_3_) δ 0.09, 0.13 (2 × s, 6H, SiMe_2_); 0.89 (s, 9H, Si*^t^*Bu); 1.30 (t, 3H, J = 7.2 Hz, –CH_3_); 1.32, 1.46 (2 × s, 6H, –CH_3_); 1.54–1.72 (m, 6H, OTHP); 3.56–3.69 (m, 1H, OTHP); 3.83–3.89 (m, 1H, OTHP); 4.15–4.36 (m, 2H); 4.55 (d, 1H, J_3,4_ = 5.5 Hz, H3); 4.66 (dd, 1H, J_2,1_ = 3.8 Hz, J_3,2_ = 9.5 Hz, H2); 4.74–4.79 (m, 2H); 4.96 (s, 1H), 5.23 (s, 1H), 5.81 (d, 1H, J_1,2_ = 3.8 Hz, H1). ^13^C NMR (75.4 MHz, CDCl_3_) δ -5.86, -4.60, 13.93, 17.88, 18.88, 25.06, 25.18, 25.15, 25.56, 26.15, 30.58, 62.44, 74.51,77.80, 79.85, 86.69, 89.78, 101.05, 101.44, 112.50, 162.32. MS(EI) (*m*/*z,%)* 504 (*M*^+^-15, 0.1), 436 (1), 190 (3), 129 (14), 85 (100); Found C 53.15, H 7.94, N 2.74 C_23_H_41_NO_10_Si requires C 53.16, H 7.95, N 2.70. The reaction scheme is shown in Fig. 2. Crystals for X-ray diffraction were obtained from ethanol.

### Refinement

H atoms were positioned geometrically with C—H = 0.96–0.98 Å and refined using a riding model, with *U*_iso_(H) equal to 1.2 (or 1.5 for methyl H atoms) times *U*_eq_(C).

### Crystal data

**Table:**

C_23_H_41_N_1_O_10_Si	*D*_x_ = 1.176 Mg m^−^^3^
*M**_r_* = 519.66	Cu *K*α radiation λ = 1.54184 Å
Orthorhombic, *P*2_1_2_1_2_1_	Cell parameters from 18 reflections
*a* = 15.593 (3) Å	θ = 17.6–42.2º
*b* = 9.563 (4) Å	µ = 1.13 mm^−^^1^
*c* = 19.690 (3) Å	*T* = 293 (2) K
*V* = 2935.9 (15) Å^3^	Prism, colourless
*Z* = 4	0.48 × 0.40 × 0.32 mm
*F*_000_ = 1120	

### Data collection

**Table:**

Enraf–Nonius TurboCAD-4 diffractometer	*R*_int_ = 0.037
Radiation source: fine-focus sealed tube	θ_max_ = 73.4º
Monochromator: graphite	θ_min_ = 3.6º
*T* = 293(2) K	*h* = −19→0
Non–profiled ω/2θ scans	*k* = −11→0
Absorption correction: ψ scan(North *et al.*, 1968)	*l* = −24→24
*T*_min_ = 0.614, *T*_max_ = 0.714	3 standard reflections
6473 measured reflections	every 167 reflections
5898 independent reflections	intensity decay: 4%
3434 reflections with *I* > 2σ(*I*)	

### Refinement

**Table:**

Refinement on *F*^2^	Hydrogen site location: inferred from neighbouring sites
Least-squares matrix: full	H-atom parameters constrained
*R*[*F*^2^ > 2σ(*F*^2^)] = 0.066	*w* = 1/[σ^2^(*F*_o_^2^) + (0.1249*P*)^2^ + 1.1254*P*] where *P* = (*F*_o_^2^ + 2*F*_c_^2^)/3
*wR*(*F*^2^) = 0.264	(Δ/σ)_max_ < 0.001
*S* = 1.11	Δρ_max_ = 0.22 e Å^−^^3^
5898 reflections	Δρ_min_ = −0.27 e Å^−^^3^
325 parameters	Extinction correction: none
Primary atom site location: structure-invariant direct methods	Absolute structure: Flack (1983), 2569 Friedel pairs
Secondary atom site location: difference Fourier map	Flack parameter: 0.04 (8)

### Special details

**Table:**

Geometry. All e.s.d.'s (except the e.s.d. in the dihedral angle between two l.s. planes) are estimated using the full covariance matrix. The cell e.s.d.'s are taken into account individually in the estimation of e.s.d.'s in distances, angles and torsion angles; correlations between e.s.d.'s in cell parameters are only used when they are defined by crystal symmetry. An approximate (isotropic) treatment of cell e.s.d.'s is used for estimating e.s.d.'s involving l.s. planes.
Refinement. Refinement of *F*^2^ against ALL reflections. The weighted *R*-factor *wR* and goodness of fit *S* are based on *F*^2^, conventional *R*-factors *R* are based on *F*, with *F* set to zero for negative *F*^2^. The threshold expression of *F*^2^ > σ(*F*^2^) is used only for calculating *R*-factors(gt) *etc*. and is not relevant to the choice of reflections for refinement. *R*-factors based on *F*^2^ are statistically about twice as large as those based on *F*, and *R*- factors based on ALL data will be even larger.

### Fractional atomic coordinates and isotropic or equivalent isotropic displacement parameters (Å^2^)

**Table:**

	*x*	*y*	*z*	*U*_iso_*/*U*_eq_	
O1	0.3708 (2)	0.3276 (4)	0.43815 (17)	0.0688 (9)	
C2	0.4611 (3)	0.3178 (7)	0.4327 (3)	0.0722 (14)	
H2	0.4895	0.3750	0.4673	0.087*	
C3	0.4804 (3)	0.1651 (6)	0.4427 (3)	0.0713 (13)	
H3	0.5281	0.1338	0.4142	0.086*	
C4	0.3965 (3)	0.0870 (6)	0.4260 (3)	0.0696 (13)	
H4	0.4041	0.0158	0.3908	0.084*	
C5	0.3381 (3)	0.2059 (6)	0.4035 (2)	0.0666 (13)	
H5	0.3447	0.2194	0.3545	0.080*	
O6	0.4953 (2)	0.1368 (5)	0.51299 (19)	0.0879 (11)	
C7	0.4381 (4)	0.0343 (7)	0.5360 (3)	0.0780 (15)	
O8	0.3698 (2)	0.0295 (5)	0.4890 (2)	0.0854 (12)	
C9	0.4844 (7)	−0.1044 (9)	0.5337 (7)	0.181 (6)	
H9A	0.4487	−0.1755	0.5534	0.271*	
H9B	0.5369	−0.0976	0.5589	0.271*	
H9C	0.4969	−0.1282	0.4874	0.271*	
C10	0.4081 (6)	0.0680 (17)	0.6031 (4)	0.194 (7)	
H10A	0.3768	0.1545	0.6017	0.290*	
H10B	0.4562	0.0774	0.6331	0.290*	
H10C	0.3712	−0.0052	0.6191	0.290*	
O11	0.4874 (2)	0.3565 (4)	0.36718 (17)	0.0756 (9)	
Si12	0.50307 (12)	0.51726 (18)	0.33863 (9)	0.0858 (5)	
C13	0.4458 (7)	0.6418 (10)	0.3943 (6)	0.166 (5)	
H13A	0.3886	0.6087	0.4023	0.249*	
H13B	0.4435	0.7318	0.3727	0.249*	
H13C	0.4757	0.6497	0.4368	0.249*	
C14	0.4610 (7)	0.5262 (12)	0.2497 (5)	0.148 (4)	
H14A	0.4664	0.4362	0.2286	0.222*	
H14B	0.4933	0.5939	0.2244	0.222*	
H14C	0.4017	0.5532	0.2506	0.222*	
C15	0.6207 (5)	0.5470 (9)	0.3385 (4)	0.107 (2)	
C16	0.6623 (6)	0.4457 (13)	0.2889 (5)	0.156 (4)	
H16A	0.7196	0.4765	0.2789	0.233*	
H16B	0.6294	0.4425	0.2478	0.233*	
H16C	0.6644	0.3541	0.3088	0.233*	
C17	0.6555 (5)	0.5320 (17)	0.4098 (4)	0.173 (6)	
H17A	0.6508	0.4363	0.4240	0.259*	
H17B	0.6231	0.5904	0.4400	0.259*	
H17C	0.7146	0.5599	0.4105	0.259*	
C18	0.6407 (8)	0.6965 (12)	0.3132 (5)	0.175 (5)	
H18A	0.7015	0.7115	0.3139	0.263*	
H18B	0.6132	0.7633	0.3424	0.263*	
H18C	0.6196	0.7075	0.2677	0.263*	
C19	0.2448 (3)	0.1860 (7)	0.4193 (2)	0.0733 (15)	
H19	0.2402	0.1481	0.4654	0.088*	
O20	0.2148 (2)	0.0833 (5)	0.37335 (18)	0.0860 (12)	
C21	0.1697 (4)	−0.0350 (9)	0.4032 (3)	0.099 (2)	
H21	0.2024	−0.0686	0.4424	0.118*	
C22	0.1651 (6)	−0.1488 (9)	0.3517 (4)	0.117 (3)	
H22A	0.1470	−0.2345	0.3738	0.141*	
H22B	0.2219	−0.1644	0.3331	0.141*	
C23	0.1036 (7)	−0.1158 (13)	0.2942 (4)	0.141 (4)	
H23A	0.1261	−0.0396	0.2670	0.169*	
H23B	0.0971	−0.1971	0.2652	0.169*	
C24	0.0191 (6)	−0.0760 (14)	0.3231 (5)	0.153 (4)	
H24A	−0.0055	−0.1549	0.3471	0.184*	
H24B	−0.0197	−0.0496	0.2869	0.184*	
C25	0.0308 (5)	0.0476 (13)	0.3724 (5)	0.143 (4)	
H25A	0.0524	0.1280	0.3476	0.172*	
H25B	−0.0242	0.0725	0.3919	0.172*	
O26	0.0894 (3)	0.0124 (7)	0.4255 (2)	0.1160 (17)	
C27	0.1881 (4)	0.3190 (8)	0.4150 (3)	0.0892 (19)	
H27	0.1297	0.2906	0.4273	0.107*	
N28	0.1840 (4)	0.3743 (8)	0.3432 (3)	0.110 (2)	
O29	0.2476 (3)	0.3920 (8)	0.3110 (3)	0.135 (2)	
O30	0.1129 (4)	0.4028 (11)	0.3218 (3)	0.194 (4)	
C31	0.2115 (5)	0.4367 (11)	0.4606 (4)	0.104 (2)	
O32	0.2205 (4)	0.5543 (8)	0.4431 (4)	0.143 (2)	
O33	0.2157 (3)	0.3927 (6)	0.5243 (2)	0.1067 (15)	
C34	0.2439 (11)	0.4994 (13)	0.5745 (5)	0.188 (6)	
H34A	0.2957	0.5446	0.5582	0.225*	
H34B	0.1998	0.5702	0.5792	0.225*	
C35	0.2591 (10)	0.4413 (15)	0.6345 (7)	0.204 (6)	
H35A	0.2092	0.3909	0.6492	0.307*	
H35B	0.2724	0.5129	0.6670	0.307*	
H35C	0.3067	0.3781	0.6308	0.307*	

### Atomic displacement parameters (Å^2^)

**Table:**

	*U*^11^	*U*^22^	*U*^33^	*U*^12^	*U*^13^	*U*^23^
O1	0.0590 (17)	0.083 (2)	0.0645 (19)	0.0041 (17)	0.0043 (15)	0.0023 (17)
C2	0.062 (3)	0.092 (4)	0.063 (3)	0.001 (3)	0.003 (2)	−0.002 (3)
C3	0.060 (3)	0.088 (4)	0.067 (3)	0.002 (3)	0.007 (2)	0.015 (3)
C4	0.067 (3)	0.082 (3)	0.059 (3)	0.001 (3)	−0.002 (2)	0.001 (3)
C5	0.054 (2)	0.093 (4)	0.053 (2)	0.000 (2)	0.0011 (19)	−0.004 (2)
O6	0.0667 (19)	0.122 (3)	0.075 (2)	−0.020 (2)	−0.0152 (18)	0.030 (2)
C7	0.072 (3)	0.092 (4)	0.070 (3)	−0.010 (3)	−0.014 (2)	0.017 (3)
O8	0.070 (2)	0.104 (3)	0.082 (2)	−0.018 (2)	−0.0152 (18)	0.032 (2)
C9	0.160 (9)	0.090 (6)	0.292 (15)	−0.005 (6)	−0.124 (10)	0.046 (7)
C10	0.115 (6)	0.37 (2)	0.091 (5)	−0.079 (10)	0.017 (5)	−0.051 (8)
O11	0.074 (2)	0.084 (2)	0.0693 (19)	−0.0028 (19)	0.0122 (17)	0.0124 (17)
Si12	0.0864 (10)	0.0890 (11)	0.0821 (9)	−0.0053 (10)	−0.0054 (8)	0.0167 (9)
C13	0.180 (10)	0.097 (6)	0.221 (12)	0.021 (6)	0.074 (9)	0.001 (7)
C14	0.161 (8)	0.161 (9)	0.123 (6)	−0.035 (7)	−0.053 (6)	0.056 (7)
C15	0.107 (5)	0.140 (6)	0.075 (4)	−0.040 (5)	0.012 (4)	0.020 (4)
C16	0.123 (7)	0.198 (11)	0.146 (8)	−0.020 (7)	0.051 (6)	−0.017 (8)
C17	0.105 (6)	0.316 (17)	0.097 (6)	−0.070 (8)	−0.027 (4)	0.043 (8)
C18	0.204 (11)	0.182 (10)	0.140 (8)	−0.114 (9)	−0.012 (7)	0.043 (7)
C19	0.055 (2)	0.118 (5)	0.046 (2)	−0.001 (3)	−0.0017 (19)	0.005 (3)
O20	0.078 (2)	0.127 (3)	0.0523 (18)	−0.021 (2)	−0.0011 (17)	0.010 (2)
C21	0.085 (4)	0.145 (6)	0.067 (3)	−0.019 (4)	−0.009 (3)	0.013 (4)
C22	0.135 (6)	0.116 (6)	0.101 (5)	−0.029 (5)	0.007 (5)	0.016 (5)
C23	0.144 (8)	0.188 (10)	0.090 (5)	−0.059 (7)	−0.017 (5)	−0.007 (5)
C24	0.112 (7)	0.228 (12)	0.119 (7)	−0.055 (7)	−0.033 (5)	0.017 (7)
C25	0.088 (5)	0.213 (11)	0.129 (7)	−0.014 (6)	−0.036 (5)	0.006 (7)
O26	0.089 (3)	0.181 (5)	0.078 (3)	−0.028 (3)	0.007 (2)	0.010 (3)
C27	0.063 (3)	0.141 (6)	0.064 (3)	0.015 (3)	0.006 (2)	0.015 (4)
N28	0.080 (3)	0.169 (6)	0.080 (3)	0.041 (4)	0.001 (3)	0.033 (4)
O29	0.096 (3)	0.208 (6)	0.101 (3)	0.043 (4)	0.023 (3)	0.067 (4)
O30	0.095 (4)	0.361 (12)	0.125 (5)	0.065 (5)	−0.021 (3)	0.084 (6)
C31	0.083 (4)	0.132 (7)	0.099 (5)	0.043 (5)	0.008 (4)	0.005 (5)
O32	0.157 (5)	0.122 (5)	0.152 (6)	0.039 (4)	−0.002 (4)	0.018 (4)
O33	0.103 (3)	0.138 (4)	0.079 (3)	0.026 (3)	0.010 (2)	−0.013 (3)
C34	0.318 (17)	0.149 (9)	0.096 (7)	0.064 (10)	−0.028 (9)	−0.043 (7)
C35	0.236 (14)	0.219 (15)	0.158 (11)	0.051 (12)	−0.025 (10)	−0.081 (11)

### Geometric parameters (Å, °)

**Table:**

O1—C2	1.415 (6)	C17—H17A	0.960
O1—C5	1.442 (6)	C17—H17B	0.960
C2—O11	1.403 (6)	C17—H17C	0.960
C2—C3	1.504 (8)	C18—H18A	0.960
C2—H2	0.980	C18—H18B	0.960
C3—O6	1.428 (6)	C18—H18C	0.960
C3—C4	1.542 (7)	C19—O20	1.414 (7)
C3—H3	0.980	C19—C27	1.552 (9)
C4—O8	1.419 (6)	C19—H19	0.980
C4—C5	1.523 (8)	O20—C21	1.456 (8)
C4—H4	0.980	C21—O26	1.403 (9)
C5—C19	1.499 (7)	C21—C22	1.488 (11)
C5—H5	0.980	C21—H21	0.980
O6—C7	1.401 (7)	C22—C23	1.517 (11)
C7—O8	1.410 (6)	C22—H22A	0.970
C7—C10	1.438 (10)	C22—H22B	0.970
C7—C9	1.511 (11)	C23—C24	1.485 (14)
C9—H9A	0.960	C23—H23A	0.970
C9—H9B	0.960	C23—H23B	0.970
C9—H9C	0.960	C24—C25	1.539 (15)
C10—H10A	0.960	C24—H24A	0.970
C10—H10B	0.960	C24—H24B	0.970
C10—H10C	0.960	C25—O26	1.429 (9)
O11—Si12	1.655 (4)	C25—H25A	0.970
Si12—C13	1.849 (9)	C25—H25B	0.970
Si12—C15	1.856 (7)	C27—C31	1.486 (12)
Si12—C14	1.871 (8)	C27—N28	1.510 (8)
C13—H13A	0.960	C27—H27	0.980
C13—H13B	0.960	N28—O29	1.188 (7)
C13—H13C	0.960	N28—O30	1.217 (7)
C14—H14A	0.960	C31—O32	1.184 (10)
C14—H14B	0.960	C31—O33	1.323 (9)
C14—H14C	0.960	O33—C34	1.487 (12)
C15—C17	1.511 (10)	C34—C35	1.327 (15)
C15—C16	1.521 (13)	C34—H34A	0.970
C15—C18	1.545 (12)	C34—H34B	0.970
C16—H16A	0.960	C35—H35A	0.960
C16—H16B	0.960	C35—H35B	0.960
C16—H16C	0.960	C35—H35C	0.960
			
C2—O1—C5	105.2 (4)	C15—C17—H17A	109.5
O11—C2—O1	110.1 (4)	C15—C17—H17B	109.5
O11—C2—C3	108.6 (4)	H17A—C17—H17B	109.5
O1—C2—C3	104.7 (4)	C15—C17—H17C	109.5
O11—C2—H2	111.1	H17A—C17—H17C	109.5
O1—C2—H2	111.1	H17B—C17—H17C	109.5
C3—C2—H2	111.1	C15—C18—H18A	109.5
O6—C3—C2	110.1 (5)	C15—C18—H18B	109.5
O6—C3—C4	104.7 (4)	H18A—C18—H18B	109.5
C2—C3—C4	105.8 (4)	C15—C18—H18C	109.5
O6—C3—H3	111.9	H18A—C18—H18C	109.5
C2—C3—H3	111.9	H18B—C18—H18C	109.5
C4—C3—H3	111.9	O20—C19—C5	106.1 (4)
O8—C4—C5	111.6 (4)	O20—C19—C27	110.2 (4)
O8—C4—C3	104.5 (4)	C5—C19—C27	115.9 (5)
C5—C4—C3	102.0 (4)	O20—C19—H19	108.1
O8—C4—H4	112.7	C5—C19—H19	108.1
C5—C4—H4	112.7	C27—C19—H19	108.1
C3—C4—H4	112.7	C19—O20—C21	116.2 (4)
O1—C5—C19	110.4 (4)	O26—C21—O20	107.8 (7)
O1—C5—C4	104.7 (4)	O26—C21—C22	114.0 (6)
C19—C5—C4	115.1 (5)	O20—C21—C22	108.5 (5)
O1—C5—H5	108.8	O26—C21—H21	108.8
C19—C5—H5	108.8	O20—C21—H21	108.8
C4—C5—H5	108.8	C22—C21—H21	108.8
C7—O6—C3	109.9 (4)	C21—C22—C23	112.8 (8)
O6—C7—O8	107.0 (4)	C21—C22—H22A	109.0
O6—C7—C10	110.3 (7)	C23—C22—H22A	109.0
O8—C7—C10	111.3 (6)	C21—C22—H22B	109.0
O6—C7—C9	107.5 (6)	C23—C22—H22B	109.0
O8—C7—C9	108.2 (6)	H22A—C22—H22B	107.8
C10—C7—C9	112.3 (9)	C24—C23—C22	109.1 (7)
C7—O8—C4	109.8 (4)	C24—C23—H23A	109.9
C7—C9—H9A	109.5	C22—C23—H23A	109.9
C7—C9—H9B	109.5	C24—C23—H23B	109.9
H9A—C9—H9B	109.5	C22—C23—H23B	109.9
C7—C9—H9C	109.5	H23A—C23—H23B	108.3
H9A—C9—H9C	109.5	C23—C24—C25	109.5 (7)
H9B—C9—H9C	109.5	C23—C24—H24A	109.8
C7—C10—H10A	109.5	C25—C24—H24A	109.8
C7—C10—H10B	109.5	C23—C24—H24B	109.8
H10A—C10—H10B	109.5	C25—C24—H24B	109.8
C7—C10—H10C	109.5	H24A—C24—H24B	108.2
H10A—C10—H10C	109.5	O26—C25—C24	110.9 (9)
H10B—C10—H10C	109.5	O26—C25—H25A	109.5
C2—O11—Si12	126.9 (4)	C24—C25—H25A	109.5
O11—Si12—C13	109.0 (4)	O26—C25—H25B	109.5
O11—Si12—C15	106.8 (3)	C24—C25—H25B	109.5
C13—Si12—C15	112.3 (5)	H25A—C25—H25B	108.1
O11—Si12—C14	108.0 (4)	C21—O26—C25	114.6 (6)
C13—Si12—C14	110.9 (6)	C31—C27—N28	108.1 (7)
C15—Si12—C14	109.8 (4)	C31—C27—C19	116.6 (5)
Si12—C13—H13A	109.5	N28—C27—C19	111.2 (5)
Si12—C13—H13B	109.5	C31—C27—H27	106.8
H13A—C13—H13B	109.5	N28—C27—H27	106.8
Si12—C13—H13C	109.5	C19—C27—H27	106.8
H13A—C13—H13C	109.5	O29—N28—O30	122.9 (6)
H13B—C13—H13C	109.5	O29—N28—C27	120.9 (5)
Si12—C14—H14A	109.5	O30—N28—C27	116.2 (6)
Si12—C14—H14B	109.5	O32—C31—O33	124.9 (9)
H14A—C14—H14B	109.5	O32—C31—C27	124.9 (8)
Si12—C14—H14C	109.5	O33—C31—C27	110.1 (8)
H14A—C14—H14C	109.5	C31—O33—C34	115.2 (8)
H14B—C14—H14C	109.5	C35—C34—O33	111.0 (12)
C17—C15—C16	112.5 (9)	C35—C34—H34A	109.4
C17—C15—C18	108.3 (8)	O33—C34—H34A	109.4
C16—C15—C18	107.3 (8)	C35—C34—H34B	109.4
C17—C15—Si12	109.8 (5)	O33—C34—H34B	109.4
C16—C15—Si12	109.0 (6)	H34A—C34—H34B	108.0
C18—C15—Si12	109.9 (7)	C34—C35—H35A	109.5
C15—C16—H16A	109.5	C34—C35—H35B	109.5
C15—C16—H16B	109.5	H35A—C35—H35B	109.5
H16A—C16—H16B	109.5	C34—C35—H35C	109.5
C15—C16—H16C	109.5	H35A—C35—H35C	109.5
H16A—C16—H16C	109.5	H35B—C35—H35C	109.5
H16B—C16—H16C	109.5		
			
C5—O1—C2—O11	76.7 (5)	C14—Si12—C15—C16	−53.3 (8)
C5—O1—C2—C3	−39.8 (5)	O11—Si12—C15—C18	−179.2 (6)
O11—C2—C3—O6	151.5 (4)	C13—Si12—C15—C18	−59.8 (7)
O1—C2—C3—O6	−90.9 (5)	C14—Si12—C15—C18	64.0 (8)
O11—C2—C3—C4	−95.9 (5)	O1—C5—C19—O20	168.6 (4)
O1—C2—C3—C4	21.7 (5)	C4—C5—C19—O20	−73.1 (5)
O6—C3—C4—O8	3.3 (6)	O1—C5—C19—C27	45.9 (6)
C2—C3—C4—O8	−113.0 (5)	C4—C5—C19—C27	164.2 (4)
O6—C3—C4—C5	119.7 (4)	C5—C19—O20—C21	127.8 (5)
C2—C3—C4—C5	3.3 (5)	C27—C19—O20—C21	−105.9 (6)
C2—O1—C5—C19	166.9 (4)	C19—O20—C21—O26	71.9 (6)
C2—O1—C5—C4	42.4 (5)	C19—O20—C21—C22	−164.1 (5)
O8—C4—C5—O1	84.2 (5)	O26—C21—C22—C23	48.8 (9)
C3—C4—C5—O1	−26.8 (5)	O20—C21—C22—C23	−71.3 (8)
O8—C4—C5—C19	−37.1 (6)	C21—C22—C23—C24	−52.3 (11)
C3—C4—C5—C19	−148.2 (4)	C22—C23—C24—C25	56.2 (11)
C2—C3—O6—C7	122.7 (5)	C23—C24—C25—O26	−58.1 (11)
C4—C3—O6—C7	9.4 (6)	O20—C21—O26—C25	69.9 (8)
C3—O6—C7—O8	−18.8 (6)	C22—C21—O26—C25	−50.6 (10)
C3—O6—C7—C10	−140.0 (7)	C24—C25—O26—C21	54.9 (10)
C3—O6—C7—C9	97.3 (6)	O20—C19—C27—C31	179.8 (5)
O6—C7—O8—C4	21.1 (7)	C5—C19—C27—C31	−59.7 (7)
C10—C7—O8—C4	141.7 (8)	O20—C19—C27—N28	−55.6 (6)
C9—C7—O8—C4	−94.4 (7)	C5—C19—C27—N28	64.9 (7)
C5—C4—O8—C7	−124.3 (5)	C31—C27—N28—O29	79.4 (9)
C3—C4—O8—C7	−14.8 (6)	C19—C27—N28—O29	−49.8 (10)
O1—C2—O11—Si12	82.7 (5)	C31—C27—N28—O30	−98.5 (9)
C3—C2—O11—Si12	−163.2 (4)	C19—C27—N28—O30	132.2 (8)
C2—O11—Si12—C13	−19.6 (6)	N28—C27—C31—O32	3.7 (10)
C2—O11—Si12—C15	101.9 (5)	C19—C27—C31—O32	129.9 (8)
C2—O11—Si12—C14	−140.2 (5)	N28—C27—C31—O33	179.9 (5)
O11—Si12—C15—C17	−60.2 (9)	C19—C27—C31—O33	−54.0 (7)
C13—Si12—C15—C17	59.2 (9)	O32—C31—O33—C34	−7.5 (12)
C14—Si12—C15—C17	−177.0 (9)	C27—C31—O33—C34	176.4 (8)
O11—Si12—C15—C16	63.5 (6)	C31—O33—C34—C35	−170.2 (11)
C13—Si12—C15—C16	−177.1 (7)		
